# The molecular characteristics of colorectal cancer: Impact of Ibuprofen and hyperthermia

**DOI:** 10.22099/mbrc.2023.45296.1802

**Published:** 2023

**Authors:** Farzaneh Zarghampoor, Behnaz Valibeigi, Abbas Behzad-Behbahani

**Affiliations:** 1 Diagnostic Laboratory Sciences and Technology Research Center, School of Paramedical Sciences, Shiraz University of Medical Sciences, Shiraz, Iran; 2 Department of Biology, Marvdasht Branch, Islamic Azad University, Marvdasht, Iran

**Keywords:** Colorectal cancer, Hyperthermia, HT29 Cells, Ibuprofen, Wnt Signaling Pathway

## Abstract

Despite various treatment options available for colorectal cancer, the survival rates for patients remain low. This study investigated the effects of hyperthermia and Ibuprofen on human colorectal adenocarcinoma cells (HT-29) viability, proliferation, and gene expression related to tumor suppression, Wnt signaling pathways, proliferation, and apoptosis The cells were exposed to hyperthermia at 42 or 43°C for 3 hours or Ibuprofen at different concentrations (700-1500 μM), and the effects were analyzed through MTT assay, trypan blue staining, and quantitative Real-time PCR. The study used quantitative Real-time PCR (qRT-PCR) to evaluate the effect of hyperthermia and Ibuprofen on the expression of various genes associated with tumor suppression, proliferation, Wnt signaling pathway, and apoptosis. The results revealed that hyperthermia caused a minor reduction in the viability and proliferation of HT-29 cells, but the decrease was not statistically significant (P<0.05). On the other hand, Ibuprofen caused a concentration-dependent decrease in the viability and proliferation of HT-29 cells. Both hyperthermia and Ibuprofen reduced the expression of *WNT1*, *CTNNB1*, *BCL2*, and *PCNA* genes, and increased the expression of *KLF4*, *P53*, and *BAX* genes. However, the changes in gene expression were not statistically significant in cells treated with hyperthermia. The findings suggest that Ibuprofen is more effective in reducing cancer cell proliferation by promoting apoptosis and inhibiting the Wnt signaling pathway than hyperthermia, which had some impact but was not statistically significant. The study highlights the potential of Ibuprofen as a targeted therapy for colorectal cancer.

## INTRODUCTION

Colorectal cancer (CRC) ranks as the third most prevalent cancer and the fourth primary cause of cancer-related fatalities worldwide. [1, 2]. Multiple factors contribute to the development of CRC, including lifestyle changes, inactivity, smoking, poor dietary habits, intestinal inflammation disease, polyps, genetics, and aging [3, 4]. Depending on the stage of CRC progression, various conventional procedures are used for treatment, including surgery, chemotherapy, and radiotherapy [5]. However, despite advances in these treatment procedures, cancer recurrences and therapeutic resistance often occur after cancer treatments; hence new targeted therapy modalities are needed to treat cancer [6]. 

Hyperthermia therapy is a new type of cancer treatment modality, which can be used alone or as an adjunct to anti-cancer treatment [7]. In hyperthermia therapy, temperatures exceeding the optimal physiological level, typically 39-45°C, are used [8]. Radiofrequency, microwave, metal nanoparticles, and laser-based hyperthermia are less or non-invasive techniques that may create heat [9, 10]. Hyperthermia affects tumor cells in different ways, including inhibition of DNA repair pathway, inhibition of systemic immune responses, denaturation of proteins, induce apoptosis, and modify essential factors of tumor survival and growth [11, 12].

Conversely, multiple studies have indicated that non-steroidal anti-inflammatory drugs (NSAIDs) are linked to a lowered risk of cancer, including breast cancer. [13], prostate [14], colorectal [15], ovarian [16], and head and neck cancers [17, 18]. NSAIDs such as isobutyl phenyl propionic acid (known as Ibuprofen) has been used for treating pain, fever, and inflammation [19]. Ibuprofen is a well-known anti-inflammatory agent that induces apoptosis, inhibits cell proliferation and angiogenesis, and enhances cellular immune responses in various types of cancer [20-22]. Therefore, the role of NSAIDs in cancer inhibition remains obscure due to controverting results.

In this study, we examined the possible anti-cancer impacts of hyperthermia and Ibuprofen on HT-29 colorectal cancer cell lines. Our focus was on determining how these treatments affect cell viability and proliferation. Moreover, the expression of tumor suppressor, Wnt signaling pathway, proliferation, and apoptosis genes was examined to understand the molecular mechanisms of anti-tumor effects of hyperthermia and Ibuprofen. 

## MATERIALS AND METHODS

**Colorectal cancer cell culture: **The HT-29 human colorectal cancer cell lines were obtained from the Pasteur Institute Cell Bank in Tehran, Iran. They were cultured in Dulbecco's modified Eagle's medium (DMEM) supplemented with 10% heat-inactivated fetal bovine serum (FBS) from Gibco, USA, and 1% penicillin-streptomycin at 37°C in a 5% CO_2_ environment. The culture medium was renewed twice a week, and when the cells reached 70-80% confluence, the plates were subcultured using 0.25% Trypsin-EDTA from Sigma, USA.

**Hyperthermia and Ibuprofen treatment** **: **To evaluate the effect of hyperthermia and Ibuprofen on colorectal cancer cells, we seeded HT-29 cells (3×104 cells/well) into 24-well plates containing DMEM supplemented with 10% heat-inactivated FBS (Gibco, USA) and 1% penicillin-streptomycin at 37°C and 5% CO_2_.

To apply hyperthermia treatment, we substituted the cell culture medium with fresh medium and incubated HT-29 cells at 42 and 43°C for 3 hours in a culture incubator with 5% CO_2_ and 95% humidity. Control cells were incubated at 37°C for 3 hours in a culture incubator with 5% CO_2 _and 95% humidity.

To treat the HT-29 cells with Ibuprofen, we aspirated the culture medium and replaced it with fresh DMEM supplemented with 3% heat-inactivated FBS and different concentrations of Ibuprofen (700, 900, 1100, 1300, and 1500 μM). Control cells were cultured with DMEM containing 10% heat-inactivated FBS and 1% penicillin-streptomycin without Ibuprofen.

**MTT assay: **To evaluate the effect of hyperthermia and Ibuprofen on cell viability, we seeded HT-29 cell lines (2×10^4^ cells/well) into 96-well plates and treated them accordingly. The untreated cells were considered the control group. After 24 and 48 hours of incubation, we replaced the medium with MTT solution (5 mg/mL; Sigma, USA) and incubated it at 37°C with 5% CO_2_ for 3 hours. Next, we centrifuged the plate at 2100 g for 5 minutes and removed the MTT solution. We added 100 μL of dimethyl sulfoxide (DMSO) to each well to dissolve the crystals, and after incubating at 37°C for 30 minutes, we measured the absorbance (570/630 nm) using a microplate reader (BioTek, USA).We determined the percentage of cell viability by comparing the optical density (OD) of treated HT-29 cells with untreated cells.

**The growth curve of HT-29 cells: **Cell proliferation was evaluated by using trypan blue staining and cell counting to determine the impact of hyperthermia and Ibuprofen. HT-29 cell lines were seeded into 24-well plates with 3×104 cells per well, and they were exposed to treatment for 24, 48, and 72 hours. A control group of HT-29 cells that did not receive any treatment was included. The cells were collected by trypsin-EDTA (0.25%) digestion and then centrifuged at 1000 g for 5 min. Subsequently, the cells were stained with trypan blue dye obtained from Sigma, USA, and counted.

**RNA extraction and cDNA synthesis:** Following exposure to hyperthermia and Ibuprofen, RNA was extracted from both treated and untreated HT-29 cells. The RNeasy Mini kit (Qiagen, Germany) was used to extract total RNA from the cells, following the manufacturer's instructions. The extracted RNA was then assessed for concentration using a nanodrop (Thermo Fisher, USA) and stored at -80°C until required. Complementary DNA (cDNA) was generated from the extracted RNA using the NG dART RT kit (EURX, Poland) according to the manufacturer's protocol. The cDNA synthesis reactions were performed using a mixture of 4 μl cDNA buffer, 1 μl primer, 1 μl NG dART RT mix, and 600 ng RNA template in a final volume of 20 μl. Amplification of DNA was carried out using a SimpliAmp Thermal Cycler (Applied Biosystem, USA) under the following cycling conditions: one cycle at 25°C for 10 min, 50°C for 50 min, and 85°C for 5 min.

**Quantitative Real-Time PCR (qRT-PCR): **To evaluate how hyperthermia and Ibuprofen affect the expression of certain genes related to tumour suppression, proliferation, Wnt signaling pathway, and apoptosis, the researchers conducted quantitative Real-time PCR (qRT-PCR) using specific primers designed through AlleleID and online primer design software such as Primer 3 and Primer-BLAST (see Table 1). The expression of the GAPDH gene was used as a control. The qRT-PCR reactions were performed using the SybrGreen PCR kit (Takara, Japan) in a final volume of 10 μl, containing 5 μl of qPCR Master Mix, 0.5 μl of each primer, 0.1 μl of ROX solution, and 2 μl of cDNA. The reactions were carried out in an ABI step one plus Real-time PCR System (Applied Biosystem, USA) under the following conditions: 95°C for 10 min, 30 cycles at 94°C for 15 s, X°C for 30 s, and 72°C for 30 s, where X°C represents the annealing temperature for each specific primer as indicated in Table 1. The experiments were performed in duplicates.

**Table 1:** Primer sequences used in qRT-PCR assay

**Name**	**Primer name**	**Primer sequence**	Tm (°C)*
*KLF4*	KF RF	5GTGCCCCGAATAACCGCTG3 5CAGGTCCAGGAGATCGTTGAAC3	62
*WNT1*	DF DR	5CGATGGTGGGGTATTGTGAAC3 5CCGGATTTTGGCGTATCAGAC3	60
*CTNNB1*	LF LR	5ACGTACAATAGCAGACACCATC3 5TCAGGGAGTCAGGGGAGG3	60
*P53*	SF SR	5TAACAGTTCCTGCATGGGCGGC3 5AGGACAGGCACAAACACGCACC3	66
*BAX*	GF GR	5CCTGTGCACCAAGGTGCCGGAACT3 5CCACCCTGGTCTTGGATCCAGCCC3	68
*BCL2*	BF BR	5TTGTGGCCTTCTTTGAGTTCGGTG3 5GGTGCCGGTTCAGGTACTCAGTCA3	64
*PCNA*	CF CR	5TAACAGTTCCTGCATGGGCGGC3 5CGTGCAAATTCACCAGAAGGC3	60
*GAPDH*	GF GR	5GGACTCATGACCACAGTCCA3 5CCAGTAGAGGCAGGGATGAT3	60

*represents the annealing temperature for each gene.

**Statistical analysis: **The qRT-PCR trials were conducted twice, while other experiments were performed three times. The findings of each test were displayed as the mean±SEM, following analysis using one-way analysis of variance (ANOVA) and the Tukey test. Statistical significance was determined by P-values that were less than 0.05.

## RESULTS

**Figure 1:**
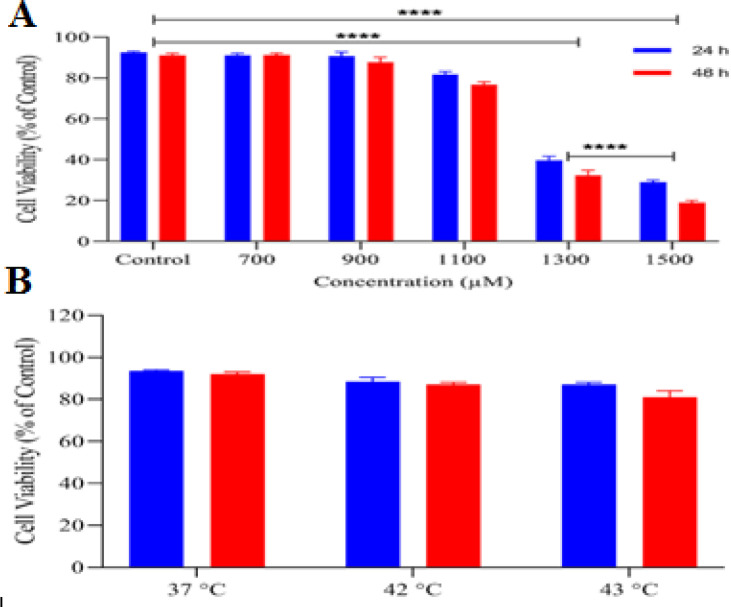
Cell viability analysis of Ibuprofen (A) and hyperthermia (B) treated HT-29 cells

The current research employed human colorectal adenocarcinoma cell lines known as HT-29 cells. The cells were adherent and had an epithelial morphology. After 72 hours, the cells underwent subculture. The MTT assay was used to assess the cytotoxic impact of Ibuprofen and hyperthermia on HT-29 cell lines. The results displayed a decrease in the viability of HT-29 cells treated with hyperthermia and Ibuprofen at concentrations of 700, 900, and 1100 μM (Fig. 1). However, the findings indicated that the combination of hyperthermia and Ibuprofen at these concentrations did not have any adverse effects on cell viability (P>0.05). Additionally, the outcomes revealed that the viability of HT-29 cell lines was inhibited by Ibuprofen at concentrations of 1300 and 1500 μM (P<0.05).

**Figure 2:**
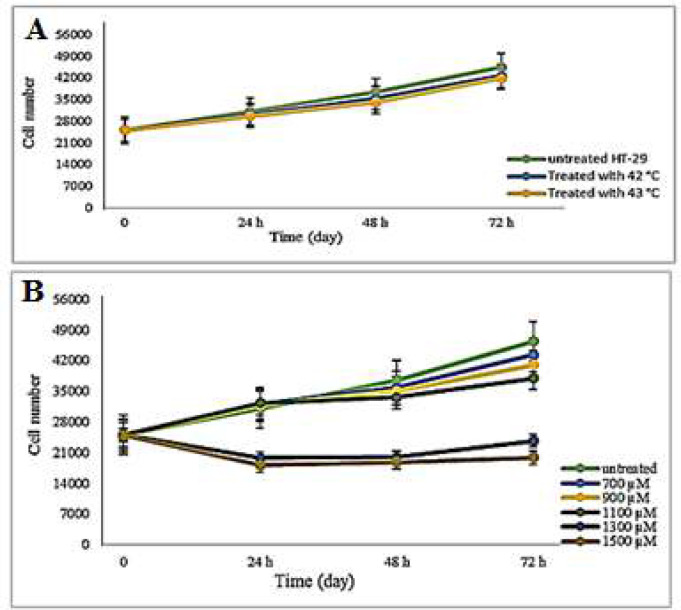
The growth curve of treated HT-29 cells with hyperthermia (A) and Ibuprofen (B) for 24, 48, and 72 hours

The study evaluated the impact of hyperthermia and Ibuprofen on cell proliferation by conducting trypan blue staining and cell counting at three different intervals (24, 48, and 72 hours). The starting number of cells used in the study was 25×10^3^ cells/well. Figure 2 indicates that there was no noteworthy difference in the proliferation of the HT-29 cells treated with hyperthermia and Ibuprofen (700, 900, and 1100 μM) as compared to the untreated cells for a period of 72 hours (P>0.05). However, the findings revealed that the proliferation of HT-29 cell lines decreased with the application of Ibuprofen with concentrations of 1300 and 1500 μM (P<0.05).

**Figure 3:**
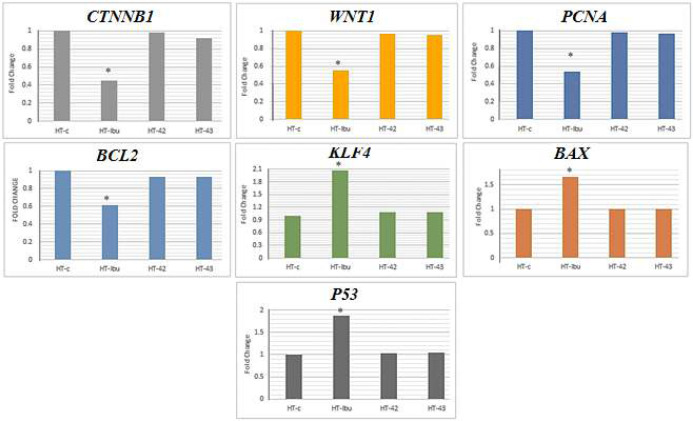
The impact of Ibuprofen and hyperthermia on the gene expression of HT-29 cells. A star (*) attached to the curve represents statistical significance at P<0.05. The vertical lines indicate the fold change observed in the gene expression levels. HT: hyperthermia, HT-c: Control group, Ibu: Ibuprofen

The study investigated the impact of hyperthermia and Ibuprofen (at a concentration of 1100 μM) on the expression of genes related to cell proliferation (*PCNA*), tumor suppression (*KLF4*), apoptosis (*P53*, *BAX*, *BCL2*), and the Wnt signaling pathway (*WNT1* and *CTNNB1*) using qRT-PCR. Results indicated that hyperthermia treatment led to reduced expression of *PCNA*, *BCL2*, *WNT1*, and *CTNNB1* genes while upregulating the expression of *P53*, *KLF4*, and *BAX* genes in HT-29 cells (Fig. 3). However, no significant differences were observed in gene expression levels between untreated and hyperthermia-treated cells at temperatures of 42°C and 43°C. Additionally, treatment with Ibuprofen led to the downregulation of PCNA, *BCL2*, *WNT1*, and *CTNNB1* gene expression, while upregulating *P53*, *KLF4*, and *BAX* gene expression in HT-29 cells, as compared to untreated cells. The observed changes were statistically significant (P<0.05).

## DISCUSSION

Although there are various modalities for treating advanced colorectal cancer, the survival rate of these patients is poor, and therapy resistance is often observed. From prokaryotes to complex cancers, cellular transporters are the main factors causing therapeutic resistance[23-25]. Other causes of therapeutic resistance are mutations in the key molecules of the signaling pathway, upregulation of anti-apoptotic proteins, and the presence of resistant tumor cells [26, 27]. Recently, NSAIDs (such as Ibuprofen) and hyperthermia have attracted attention as modalities that might benefit the treatment of numerous cancers [10, 28]. 

Therefore, we examined how hyperthermia and Ibuprofen affect colorectal cancer cells. Initially, we assessed their impact on the growth and survival of HT-29 cells. Our findings suggested that hyperthermia led to a decrease in the growth and survival of treated HT-29 cells. Nevertheless, these changes were not significantly different from those observed in untreated HT-29 cells. Furthermore, our outcomes demonstrated that the decrease in the growth and survival of Ibuprofen-treated HT-29 cells was dependent on the drug concentration. These findings were similar to those reported in earlier studies on cancer cell lines from the breast, prostate, ovary, and kidney [29, 30]. 

One of the signaling pathways that play a critical role in the development of various cancers, particularly CRC, is the Wnt/β-catenin signaling pathway [31]. Aberrant activation Wnt/β-catenin signaling pathway due to genetic alterations leads to resistance to various conventional therapies by maintaining cancer stem cell populations, enhancing DNA damage repair, and facilitating transcriptional plasticity [32]. Investigations have also demonstrated that Wnt/β-catenin signaling could facilitate tumour chemoresistance through the inhibition apoptosis pathway [33]. Numerous studies demonstrated that NSAIDs such as Ibuprofen decreased cell viability and proliferation, and induced morphological changes and apoptosis [34]. Our results revealed that Ibuprofen and hyperthermia reduced the expression of the key molecules of the Wnt/β-catenin signaling pathway (*WNT1* and *CTNNB1*). Nevertheless, downregulation of expression of *WNT1* and *CTNNB1 *genes was not significantly in the hyperthermia-treated HT-29 cell lines compared with untreated HT-29 cell lines. Furthermore, we found that HT-29 cells treated with Ibuprofen had a reduced expression of *PCNA*, possibly due to a decreased Wnt/β-catenin signaling pathway. Alternatively, a reduction in *PCNA* expression could also indicate a decline in cell proliferation.

Moreover, an increase was observed in the expression of the Kruppel-like factor 4 (*KLF4*) gene in the hyperthermia and Ibuprofen treatment HT-29 cells. KLF4 is an epithelial transcription factor that might be played the role of a tumour suppressor or an oncogene depending on the context of tumours and is downregulated in many colorectal cancers [35, 36]. It has shown that the increased KLF4 marker as a tumour suppressor can successfully suppress the proliferation or migration of colorectal cancer cells [37, 38], which was also in agreement with the results of the present study. Besides, evidence has shown that the KLF4 marker interacts with β-catenin and represses Wnt signaling pathway [39]. 

The escape from the apoptosis signaling pathway and the upregulation of anti-apoptotic proteins are among the leading causes of therapeutic resistance. In this study, we found that Ibuprofen and hyperthermia treatment increased the expression of *P53* and *BAX* genes while decreasing the expression of the *BCL2* gene, which aligns with previous reports that Ibuprofen induces apoptosis in prostate and microglia cell lines. These findings suggest that Ibuprofen promotes apoptosis at the molecular level by upregulating the apoptosis pathway. The increased transcription level of the p53 tumor suppressor gene may have induced apoptosis through the BCL2/BAX pathway, while inhibiting cell proliferation through PCNA expression. Additionally, the KLF4 marker may increase the DNA-recognition specificity binding of P53 to specific locations *in vivo*, and induce apoptosis through increased BAX and decreased BCL2 expression, as many studies have demonstrated [40-42]. 

### Acknowledgements:

The present article was extracted from a Ph.D. thesis and supported by the Department of Biology, Marvdasht Branch, Islamic Azad University, Marvdasht, Iran, and Department of Medical Physics, School of Medicine, Shiraz University of Medical Sciences, Shiraz, Iran. The study team would like to gratefully acknowledge the staff of these centers for their sincere cooperation.

### Conflict of Interest:

None
